# Screening of LAB strains and their co-culture fermentation with *Bacillus subtilis* of Cili fruit substrate: impact on γ-aminobutyric acid enrichment, key enzyme activities, bioactive and functional properties

**DOI:** 10.3389/fnut.2025.1622745

**Published:** 2025-07-04

**Authors:** Angelo Uriho, Jacob Ojobi Omedi, Cheng Chen, Kaiwen Chen, Shuning Zhang, Li Liang, Yan Xu, Ning Li, Weining Huang

**Affiliations:** ^1^State Key Laboratory of Food Science and Resources, and The Laboratory of Baking and Fermentation Science, Cereals/Sourdough and Nutritional Functionality Research, School of Food Science and Technology, Jiangnan University, Wuxi, China; ^2^Key Laboratory of Industrial Biotechnology of Ministry of Education, School of Biotechnology, Jiangnan University, Wuxi, China; ^3^Guangzhou Puratos Food Co., Ltd., Guangzhou, China

**Keywords:** Cili fruit, co-culture fermentation, lactic acid bacteria, *Bacillus subtilis*, γ-aminobutyric acid

## Abstract

In this study, 20 lactic acid bacteria (LAB) strains were screened for glutamic acid decarboxylase activity (GAD); and used as co-culture starters with *Bacillus subtilis* to enrich the γ-Aminobutyric acid (GABA) content in Cili fruit. Changes in physiochemical properties, key enzyme activities, bioactive composition and functional properties in the co-culture fermented Cili fruit substrate was reported. Five of 20 LABs exhibited adequate GAD activity, but only three strains (*Levilactobacillus brevis* DS4-15: BsLb, *Lactiplantibacillus plantarum* N2-9: BsLp, *Limosilactobacillus fermentum* BT2-3: BsLf), enhanced the GABA content in Cili fruit at the optimized co-culture fermentation conditions. Compared to *B. subtilis* fermented substrate (Bs), a significant decrease in pH (4.89, except BsLf), increase in TTA (19.30–29.20 mL) was observed after co-culture fermentation of Cili fruit. Moreover, higher GABA (102.48–585 mg/kg), total phenol (256–275.17 GAE/g), total flavonoid (140.54–172.33 QE/g), and total free amino acid (2,278.37–6,191.39 mg/kg FW), but decreased vitamin C (740.48–960.59 mg/kg FW) content was observed after co-culture fermentation, especially in the order BsLf, then BsLp, and BsLb. Subsequently, the antioxidant activity and anti-hangover properties increased in BsLf, BsLp and BsLb compared to Bs and unfermented Cili fruit. The changes observed were attributed to LAB strains’ ability to alter pH during co-culture fermentation with *B. subtilis* which was optimal for increased enzyme activities of esterase, β-glucosidase, GAD, protease and ascorbate oxidase during co-culture fermentation (BsLf > BsLp > BsLb). This increased the release of bioactive metabolites in Cili fruit and enhanced its functional properties. These findings reveal that there was a synergy in co-culture fermentation which improved the bioactive and functional properties of Cili fruit as a novel GABA enriched ingredient for potential use in the food industry.

## 1 Introduction

Cili (*Rosa roxburghii* Tratt) fruits, also known as chestnut rose, are traditional fruits widely consumed for their medicinal purposes in Southwest China. Nutritionally, Cili fruit, contained several components such as ascorbic acid, amino acids (including, γ-aminobutyric acid [GABA]) and polyphenols (1, 2) which contributed to its enhanced functional and health promoting properties (3, 4). The content of GABA, a nonprotein amino acid which was crucial in the prevention and treatment of various diseases and disorders such cardiovascular diseases, depression, anxiety, and Alzheimer’s disease (5, 6), was significantly higher in Cili fruit (112.17 mg/kg FW) (7) than other fruits such as strawberries (15–35 mg/kg FW) (8), grapes (47–74 mg/kg FW) (9), and lemons (70 mg/kg FW) (10). However, the content of GABA naturally present in plant foods, including Cili fruit, was still considered very low (11).

During microbial fermentation of fruit substrates, diverse nutritional and functional properties are elevated after fermentation (12, 13). For instance, the content of phytochemicals, total amino acids and GABA of Cili fruit were enhanced after fermentation using lactic acid bacteria (LAB) and other probiotic strains as starters (14–17). In addition, compared to chemical methods, fermentation using carefully selected microorganisms may offer a cleaner and sustainable approach to enriching the GABA content of plant foods such as Cili fruit (18).

Microorganisms used as starters for GABA enrichment in several substrates have been shown to exhibit high glutamic acid decarboxylase (GAD), an enzyme that facilitates the conversion of glutamic acid to GABA during fermentation (19). Several species of *Bacillus* such as *B. subtilis* and *B. coagulans*, commonly known for their probiotic properties such as heat-resistance and ability to grow in the harsh gastrointestinal tract (20, 21), have been reported to be good GABA producers during substrate fermentation (22). On the other hand, LAB of genera *Lactobacillus*, *Leuconostoc*, *Weissela*, and *Pediococcus* are generally regarded as safe and have been commonly used as starters in diverse food fermentations (23). In recent years, several LAB strains with GABA producing ability have been reported (24). Moreover, the LAB strain’ GABA producing abilities were influenced by substrate, temperature, pH, and co-factors such as L-sodium hydrogen glutamate (L-MSG) and pyridoxal phosphate (PLP), among others (24, 25). Furthermore, the LAB strains’ ability to lower the substrate pH to that for the optimal GAD enzyme could further promote its activity leading increased GABA content in the fermented substrate (26). However, few known studies have explored the influence of co-culture fermentation of *Bacillus* spp. and LAB strains starters on the GABA enrichment of Cili fruit.

Therefore, the objective of this study was to investigate the effect of co-culture fermentation using *Bacillus subtilis* and LAB strains previously isolated from traditional sourdough starters collected from different regions in China on GABA enrichment of Cili fruit. Twenty LAB strains belonging to *Lactiplantibacillus plantarum*, *Levilactobacillus brevis*, *Pediococcus pentosaceus*, and *Limosilactobacillus fermentum* were selected and screened for GAD enzyme activity. The LAB strains with acceptable GAD activity were selected and used as co-culture starters with *Bacillus subtilis* BNCC190341 to enrich the GABA content in Cili fruit substrate, and the fermentation conditions were optimized by using Response Surface Methodology. Changes in pH, TTA and key enzymatic activities (e.g., esterase, β-glucosidase, GAD, protease, and ascorbate oxidase) during the co-culture fermentation were also reported. Furthermore, the bioactive composition (e.g., Vitamin C, total phenol and flavonoid content, and free amino acids) and functional properties (e.g., antioxidant activity, anti-hangover) of the co-culture fermented Cili fruit were determined.

## 2 Materials and methods

### 2.1 Materials

Sundried Cili (*Rosa roxburghii* Tratt) fruits were purchased from Guiding County Zhou Xigeng Special Food (Guizhou, China). Naturally sun-dried Cili fruit was milled whole into a fine powder using an electric stainless-steel grinder (DAMAI, Wuyi Haina Electric Appliance Co., Ltd., Zhejiang, China). The powder was packed in the polythene bags and stored at freezing temperature (−80°C) for further use. Monosodium glutamate (MSG), γ-aminobutyric acid (GABA), and pyridoxal phosphate (PLP) were purchased from Shanghai Yuanye Biotechnology Co., Ltd. (Shanghai, China). All other chemicals and reagents used were of analytical grade.

### 2.2 Microorganism culture media and growth conditions

Twenty LAB strains, including, *Levilactobacillus brevis*: AT1-4, BS1-3, BS2-4, DT2-6, DS4-15, *Lactiplantibacillus plantarum*: AS0-1, AS1-1, AT2-2, AS2-4, N2-9, *Pediococcus pentosaceus*: BS0-3, BS2-10, BS0-14, BT1-1, BT1-4, and *Limosilactobacillus fermentum*: BT2-2, BT2-3, BT2-3, Y’C-22, 8′-2 previously isolated from Qu starters from different regions of China (A: Zhumadian Henan, B: Nanyang, C: Xingtai Hebei, D: Linfei Shanxi, N: Neimenggu Inner Mongolia, Y: Yinchuan Ningxia), were obtained from the Laboratory of Baking and Fermentation Science, Cereals/Sourdough and Ingredient Functionality Research, Jiangnan University. *Bacillus subtilis* subsp. subtilis BNCC190341 was purchased from Beina Chuanglian Biotechnology Co., Ltd. (Henan, China). For use, the LAB strains were activated in Man Rogosa Sharpe (MRS) broth at 37°C overnight under anaerobic conditions, then transferred into fresh broth and cultured to late exponential phase (18 h). For the *B. subtilis* BNCC190341, it was grown in Luria–Bertani (LB) broth at 37°C and 150 rpm for 18 h under aerobic conditions.

### 2.3 Qualitative screening of GABA producing activity of LAB strains

For GABA synthesizing ability, bromocresol green was used as a pH indicator for GAD activity in the different LAB strains as described by Edalatian et al. (27). Briefly, overnight cultures were washed once with 5 mL of 0.85% (w/v) NaCl solution and centrifuged (5,000 g, 20 min, 25°C). The bacterial pellets obtained were dissolved in 500 μL of test solution (pH 3.5) (containing 1 g of L-glutamic acid, 90 g NaCl, 300 μL of Triton X-100, and 0.05 g bromocresol green dissolved in 1 L of water). GAD activity was determined visually through color change as described by Edalatian et al. (27). The LAB strains which exhibited GAD activity (low to high) were selected.

### 2.4 Effect of simulated gastrointestinal tract (GIT) conditions and heat on the selected LAB strains and *B. subtilis*

#### 2.4.1 Tolerance to artificial gastric and intestinal fluids

Tolerance to artificial gastric and intestinal fluids was determined following the method described by Liu et al. (28) with some modifications. The artificial gastric juice was prepared using 3 mg/mL pepsin dissolved in sterile isotonic phosphate-buffered saline (pH 7.4) adjusted to pH 2.5 with 0.1 mol/L hydrochloric acid. The resultant artificial gastric fluid was subsequently filtered via a 0.22-μm membrane (China Hefei Biosharp Biotechnology Co., Ltd.) to ensure sterilization before utilization. The artificial intestinal fluid was prepared with 1.0 g/L of trypsin (Shanghai Yuanye Biological Technology Co., Ltd., Shanghai, China) and 1.8% bile salt (Sinopharm Chemical Reagent Co., Ltd., Shanghai, China) dissolved in sterile PBS and adjusted to pH 8.0 with 0.1 mol/L sodium hydroxide. The resultant artificial intestinal fluid was filtrated using a 0.22-μm membrane filter (China Hefei Biochemical Co., Ltd.) for sterilization before use.

For use, the bacterial pellets obtained from activated selected LAB strains or *B. subtilis* after centrifugation (5,000*g*, 10 min, 4°C), were rinsed twice and re-suspended in 5 mL sterile phosphate-buffered saline (PBS) (pH 7.4). For resistance to artificial gastric juice, 0.5 mL containing 8.02 Log CFU/mL counts of each bacterial culture was added to 4.5 mL of artificial gastric fluid (pH 2.5) and incubated at 37°C under anaerobic conditions. Samples were collected at 0 and 3 h, respectively, and the live bacteria were enumerated on the respective culture plates. After the 3 h in the artificial gastric juice, 0.5 mL of each sample was transferred into artificial intestinal juice (4.5 mL), followed by incubation (37°C) under anaerobic conditions. The viable cell counts of the bacteria was determined after 4 and 8 h. The results were reported as colony-forming units per milliliter (Log CFU/mL) from the viable cell count.

#### 2.4.2 Heat resistance

The thermotolerant capacity of the selected LAB strains and *B. subtilis* was determined according to methods by Pérez-Chabela et al. (29) and Tu et al. (30), respectively, with some modifications. Each strain was cultured in 5 mL MRS broth and incubated at 37°C for 18 h. The tubes were immersed vertically in a water bath at three different temperatures, including 45°C to simulate conditions such as sun-drying, warm fermentation or prolonged storage in tropical climates, 85°C to simulate mild thermal processing, and 100°C to simulate boiling, canning, or retort sterilization, for 10 min. After the different thermal treatments, the viable cell counts were determined by serially dilution and plating the appropriate dilutions on MRS agar for LAB strains and LB agar for *B. subtilis* for 24–48 h at 37°C.

### 2.5 Co-culture fermentation of Cili fruit and optimization by using response surface design methodology

Cells from *B. subtilis* were mixed with each of the selected LAB strains and used as co-culture starters in the co-culture fermentation of Cili fruit. The co-culture fermentation conditions of 3 independent factors, including, MSG (1.32–4.68%), PLP (0.01–0.25 mM), and fermentation time (48-96 h) were varied using the response surface design methodology (Design Expert, V.8.0.6). The Cili fruit powder was mixed with water at a 20:80 (w/v) ratio. Twenty treatment combinations were generated under the central composite design and tested (Supplementary Table S1). Co-culture fermentation was carried out in the 250 mL Erlenmeyer flasks using a shaking incubator (150 rpm) at a constant temperature of 37°C. The total fermentation time based on the condition generated by the central composite design was split into two stages. The first stage was aerobic fermentation with 3% *Bacillus subtilis* starter (8 log CFU/g) for 24 h sealed with breathable films. The remaining total time (72 h), a second stage based on anaerobic fermentation with 3% LAB starter (8 log CFU/g), was done after sealing the fermentation containers. The GABA content was used to select the optimized fermentation condition. The GABA content at the optimized co-culture fermented Cili fruit was determined according to the method described by Saraphanchotiwitthaya and Sripalakit (31) with some modifications. A 100 μL sample obtained from supernatant extracts of fermented Cili fruit, was derivatized with 500 μL of OPA-MCE and vortexed for 2 min before analysis. All samples and the mobile phase were filtrated using 0.22 μm syringe filter discs and degassed via sonication. Derivatized samples (20 μL) were injected into the Alliance HPLC system (Waters, Milford, MA, USA) equipped with a 2,695 separation module, 2,998 PDA detector and Waters Symmetry C18 column (5 μm, 250 × 4.6 mm; Waters, USA) at a 1 mL/min flow rate, and the column temperature was 35°C. The mobile phase consisted of a 0.1 M sodium acetate buffer and methanol in a 70:30 (v/v) ratio, with a pH of 7.2. A UV detector at 254 nm identified the OPA derivatives of GABA. The calibration curve was established using a GABA standard concentration of 125–1,000 μg ml^−1^, with a coefficient of determination (*R*^2^) above 0.99.

### 2.6 Changes in pH and the total titratable acidity (TTA) during the co-culture fermentation of Cili fruit

The changes in TTA, pH and microbial cell counts was done on samples collected at 0, 18, 36, 54, and 72 h periods during the co-culture fermentation process of Cili fruit. The TTA and pH was determined in 10 g of sample thoroughly mixed in 90 mL of distilled water using a magnetic stirrer. The pH was measured using a pH meter (FE-20, Mettler Toledo, Shanghai, China), while the TTA was expressed as the mL of 0.1 N NaOH needed to achieve a pH of 8.5.

### 2.7 Changes in key enzyme activities during the co-culture fermentation of Cili fruit

Supernatants from sample extracts aseptically collected at 0, 18, 36, 54, and 72 h of the co-culture fermentation period were centrifuged (10,000×*g*, 15 min, 4°C) and used to assess the enzyme activity.

#### 2.7.1 Esterase enzyme activity

Esterase activity was determined using the method described by Gao et al. (32) with some modifications. Briefly, sample extract (200 μL) was mixed with 80 μL of 25 mM p-NP-butyrate in 1.72 mL of 0.1 M citrate buffer (pH 5.0) and incubated at 37°C for 60 min. Thereafter, 0.2 mL of 0.5 M NaOH was added to terminate the reaction. The p-nitrophenol (p-NP) released was measured at 410 nm using a UV–visible spectrophotometer (Harbor City, CA, USA) compared to a blank prepared without sample extracts. One unit of enzyme activity was defined as amount of enzyme required to release l μmol p-NP from p-NP-butyrate under the above conditions.

#### 2.7.2 β-Glucosidase enzyme activity

β-glucosidase activity was evaluated by quantifying the amount of p-NP liberated from 4-nitrophenyl β-D-glucopyranoside (p-NPG) as substrate in a method described by Liang et al. (33) with some modifications. Briefly, to 100 μL of sample extract, 1.8 mL of 0.05 M sodium acetate buffer (pH 5) was added and incubated at 37°C for 5 min, after which 100 μL of substrate p-NPG was added and incubated at 37°C for 10 min. The reaction was terminated by adding 1 mL of 1 M Na_2_CO_3_, and the optical density at 400 nm was recorded. One enzyme unit was defined as amount of enzyme required to catalyze production of 1 μmol of p-NP per minute under the above conditions.

#### 2.7.3 Glutamate decarboxylase (GAD) enzyme activity

The *in vitro* GAD enzyme activity was determined by directly measuring the GABA produced after GAD reaction as described by Lü et al. (34) with some modifications. Briefly, 1 g of sample extract was incubated with 5 mL of reaction mixture (containing 50 mM sodium phosphate (pH 5.8), 100 mM L-glutamate, 0.2 mM pyridoxal 5′-phosphate) at 40°C for 60 min, then the reaction was terminated in a water bath at 90°C for 5 min. After centrifugation (3,000*g*, 10 min), it was derivatized with OPA-MCE solution and filtered, and the GABA in the supernatant was assayed. One unit of GAD activity was defined as 1 mM of GABA produced from glutamate per gram of sample extract.

#### 2.7.4 Protease enzyme activity

The enzymatic activity of the protease was measured indirectly by the colorimetric reaction of the protease decomposition of casein (substrate) to produce phenolic amino acids (35). One milliliter of 1% tyrosine solution and 1 mL supernatant were preheated for 5 min at 40°C and then reacted in a test tube in a water bath at 40°C for 10 min. Two milliliters of 0.4 mol/L trichloroacetic acid were immediately added successively. It stood for 10 min and was filtered with a slow qualitative filter paper. One milliliter of the filtrate was successively added with 5 mL Na_2_CO_3_ solution and 1 mL Folin–Ciocalteu reagent, and the absorbance value was measured at 680 nm after 20 min in a water bath at 40°C. Total protease activity was expressed in units (U/mL).

#### 2.7.5 Ascorbate oxidase enzyme activity

The activity of ascorbate oxidase was measured using the method described by Leong et al. (36). The reaction assay was a mixture of 2,500 μL of sodium phosphate buffer (0.1 M, pH 6.0) containing 0.5 mM EDTA, 400 μL of sample extract, and 100 μL of L-AA substrate solution (0.5 mM) L-AA dissolved in sodium phosphate buffer (0.1 M, pH 5.6) containing 0.5 mM EDTA. The absorbance was measured in a spectrophotometer at 265 nm (ε265 = 14.3 mM^−1^ cm^−1^). The blank solution was a mixture of 2,600 μL sodium phosphate buffer (0.1 M, pH 6.0), containing 0.5 mM EDTA, and 400 μL of carrot extract. One unit of AAO activity was defined as the amount of enzyme AAO that catalyzed the oxidation of 1 μmol of L-AA to DHAA per minute at 25°C and pH 6.0.

### 2.8 Effect of co-culture fermentation on the bioactive composition of Cili fruit

#### 2.8.1 Vitamin C content

Vitamin C content was determined following a method described by Uriho et al. (37) with some modifications. Briefly, 20 mL of 4.5% metaphosphoric acid was mixed with 5 g of samples, then centrifuged (6,000*g*, 15 min, 10°C), and filtered through a 0.22 μm syringe filter. Ten microliter of the supernatant was injected into the HPLC system using a mobile phase consisting of 3, 30 and 970 mL of formic acid, methanol and water, at a flow rate of 1 mL/min, using T3 column (4.6 × 250 mm, 5 μm, Waters, USA), and a 2,998 Photodiode Array (PDA) detector to detect light at a wavelength of 245 nm. The column was maintained at a constant temperature of 35°C.

#### 2.8.2 Total phenolic (TPC) and flavonoid (TFC) content

The TPC was determined in methanol sample extracts using the Folin–Ciocalteu method as described by Omedi et al. (38) with some modifications. Briefly, 5 mL of distilled water, 0.2 mL of methanol sample extracts, and 0.5 mL of Folin–Ciocalteu reagent were combined. After 3 min, 1.0 mL of saturated Na_2_CO_3_ solution (37%, w/v) was introduced, and the mixture was stirred. The volume was modified using distilled water to 10 mL, and the flask was placed in darkness for 1 h at ambient temperature. Absorbance was recorded at 760 nm using a blank of distilled water in place of the methanol extract. The TPC was determined using a standard curve of gallic acid (0–50 μg/mL) as a reference. Results were presented as milligrams of gallic acid equivalents per gram of sample (mg GAE/g).

The TFC was determined by the AlCl_3_–NaNO_2_ colorimetric method as described by Zhang et al. (39). Briefly, 150 μL of methanol extracts was reacted with 1 mL NaNO_2_ (5%, m/v) for 3 min. Thereafter, 1 mL of 0.27 mol/L aluminum nitrate solution, then 4 mL of 1 mol/L NaOH solution was added into the mixture. Absorbance at 502 nm was recorded after 15 min of reaction at ambient temperature. Rutin was used as the standard. The TFC was expressed as mg of quercetin equivalents (QE) per gram of sample (mg QE/g).

#### 2.8.3 Free amino acid (FAA) content

The FAA content in the fermented Cili fruit was determined by chromatographic methods as described by Omedi et al. (40) with some modifications. Briefly, 1 g of sample was added to 25 mL of 5% (w/v) TCA and ultrasound treated for 20 min, followed by centrifugation (15,000*g*, 30 min, 4°C). The supernatants were then filtered using a 0.22 μm filter before injection into HPLC for analysis. The amino acid profiles were analyzed using liquid chromatography (Ag 1100, Agilent America) with UV detection following OPA/FMOC pre-column derivatization. Chromatographic separation was conducted using the ODS HYPERSIL column (5 μm, 250 mm × 4.6 mm) from Agilent America. The mobile phase A comprised 6.5 g of crystalline sodium acetate dissolved in 1,000 mL of water, to which 225 μL of triethylamine was incorporated and mixed. Acetic acid (5%) was drop-wise added to achieve a pH of 7.2, followed by adding 5 mL of butylene oxide, and the solution was subsequently mixed. Mobile phase B: 6.5 g of sodium acetate crystals were dissolved in 400 mL of water, and 5% acetic acid was drop-wise added to achieve a pH of 7.2. The solution was thoroughly combined with 800 mL of acetonitrile and 800 mL of methanol. The elution gradient for amino acid analysis was as follows: at 0 min, B% was 8; at 20.1 min, B% was 50; and at 24 min, B% was 100. The flow rate was 1.0 mL/min, utilizing a DAD (diode array detector) UV detector with wavelengths ranging from 338 nm to 262 nm (Pro, Hypro), with a column temperature set at 40°C. Amino acids were quantified using an external standard method.

### 2.9 Effect of co-culture fermentation on functional properties of Cili fruit

#### 2.9.1 DPPH, ABTS, and FRAP antioxidant activity

The DPPH radical scavenging capacity was evaluated following the method described by Zhao et al. (41) with some modifications. Briefly, 100 μL of methanolic extracts were added to 1.9 mL of 186 μmol/L DPPH. The mixture was allowed to stand for 30 min at ambient temperature. Absorbance of the mixture was measured using a UV–visible spectrophotometer (UV-1800, Shimadzu, Kyoto, Japan) at a wavelength of 517 nm.

The ABTS radical scavenging activity was assessed following the method described by Yin et al. (42) with some modifications. Briefly, a solution comprising 7.4 mM ABTS and 2.6 mM K_2_S_2_O_8_ was prepared by combining the two substances without light for 12 h. The ABTS + solution was diluted and mixed with either the test sample or buffer at a volume ratio of 7:1. The resultant mixture was kept in darkness for 6 min, after which absorbance was measured at wavelength of 729 nm.

The FRAP was conducted following the method described by Ekumah et al. (43) with some modifications. Briefly, 3.8 mL FRAP reagent (mixture of 10 parts 300 mM sodium acetate buffer (pH 3.6), 1-part 10 mM TPTZ (2,4,6-tripyridyl-s-triazine) in 40 mM HCl and 1-part 20 mM FeCl_3_·6H_2_O) was added to 0.2 mL test sample. The resulting solution was incubated for 30 min at 37°C, and the absorbance was measured at 593 nm using a UV–vis spectrophotometer (UV-1800, Shimadzu, Kyoto, Japan). The results were expressed in milligram equivalents of FeSO_4_ per mL of sample using a FeSO_4_ standard curve generated under the same conditions.

#### 2.9.2 Anti-hangover activity

The alcohol dehydrogenase (ADH) activity was determined following the Valle & Hoch method (44). Briefly, 1.5 mL pyrophosphate buffer (0.1 M, pH 8.8), 0.1 mL of 0.25 U/mL ADH, 0.5 mL ethanol (11.5%, v/v), and 0.1 mL of fermented Cili fruit sample were mixed at 25°C, followed by addition of 1.0 mL 0.01 M NAD + to initiate the reaction. Absorbance was determined at 340 nm by a microplate reader and was measured again after 15 min.

The hydroxyl scavenging activity was determined using the method described by Zhou et al. (45) with some modifications. For the color reaction of ferric sulfate and salicylic acid, 1 mL 9 mmol/L FeSO_4_ solution was mixed with 1 mL 9 mmol/L salicylic acid ethanol solution, and then 8.8 mmol/L H_2_O_2_ solution (0.1 mL) was added. After shaking, the sample solution (0.1 mL) was added, and absorbance was measured at 510 nm.

### 2.10 Statistical analysis

Results of three independent assays were presented as mean values. Data was compared using one-way analysis of variance (ANOVA), while multiple comparisons of data was performed by Tukey’s test at *p* < 0.05 level of significance using SPSS 16.0 (SPSS Inc., Chicago, IL, USA).

## 3 Results and discussion

### 3.1 Screening of GABA producing lactic acid bacteria (LAB)

Several studies have demonstrated that some *B. subtilis* and various LAB strains are capable of producing GABA through the GAD enzyme action which catalyzes the decarboxylation of glutamic acid to GABA (46). In this study, the GAD activities of LAB strains were used to estimate their GABA producing ability and the screening results were presented in Table 1. Of the 20 LAB strains, 2 had medium (+++, greenish blue), 3 had low (++, green), 13 had very low (+, greenish yellow), and 2 had no (−, yellow) GAD activity (Table 1; Supplementary Figure S1). Therefore, 5 LAB strains: AS1-1, N2-9 (*Lactiplantibacillus plantarum*); 8′-2, BT2-3 (*Limosilactobacillus fermentum*); and DS4-15 (*Levilactobacillus brevis*) were selected as starters in the subsequent experiments.

**Table 1:** Screening of GABA producing LAB strains based on GAD activity and color changes.

Strain	Strain code	% Activity	GAD activity*	Color change
*Levilactobacillus brevis*	AT1-4	0	−	Yellow
	BS1-3	25	+	Greenish yellow
	BS2-4	25	+	Greenish yellow
	DT2-6	25	+	Greenish yellow
	DS4-15	50	++	Green
*Lactiplantibacillus plantarum*	AS0-1	0	−	Yellow
	AS1-1	50	++	Green
	AT2-2	25	+	Greenish yellow
	AS2-4	25	+	Greenish yellow
	N2-9	75	+++	Greenish blue
*Pediococcus pentosaceus*	BS0-3	25	+	Greenish yellow
	BS2-10	25	+	Greenish yellow
	BS0-14	25	+	Greenish yellow
	BT1-1	25	+	Greenish yellow
	BT1-4	25	+	Greenish yellow
*Limosilactobacillus fermentum*	BT2-2	25	+	Greenish yellow
	BT2-3	50	++	Green
	D23	25	+	Greenish yellow
	Y’C-22	25	+	Greenish yellow
	8′-2	75	+++	Greenish blue
*Bacillus subtilis*	190,341	100	++++	Blue

*Qualitative GAD activity based on colorimetric results, where: (++++) represented high GAD activity (Blue), (+++) represented medium GAD activity (Greenish blue), (++) represented low GAD activity (Green), (+) represented very low GAD activity (Greenish yellow), and (−) represented no GAD activity (Yellow).

### 3.2 Tolerance of the selected LAB strains to gastrointestinal conditions and heat

The changes in the tolerance to gastrointestinal conditions and heat by the selected LAB strains was presented in Table 2. The results showed that the viability of *B. subtilis* was not affected after exposure to the gastric and intestinal solution, and the different heat treatments. This was in agreement with studies that demonstrated that *B. subtilis* could withstand and survive in the harsh environment of the GI tract characterized by low pH levels, and high temperatures (47). In one study (48), *Bacillus subtilis* viability remained unaffected in gastric juice (pH 2.0) for 3 h, while retaining more than 90% of its viability in intestinal juice (pH 8.0) after 24 h. Furthermore, due to the ability of *Bacillus* strains to form spores (30), the *B. subtilis* used in this study could withstand high temperatures. On the other hand, the viability of the LAB strains in this study generally decreased in the range of 17.04–20.38% in gastric juice and 29.43–34.23% in intestinal juices; with more than half or total viability loss in all LAB strains after 85°C and 100°C heat treatment (Table 2). This indicated that the despite the decrease in their viability due to sensitivity to low pH environment, the remaining viable LAB strain cells adequately survived the stomach (6.25–6.74 Log CFU/mL) and intestinal (5.47–5.59 Log CFU/mL) conditions (Table 2) (49). However, the source of LAB strains and their strain differences may have influenced their poor thermal tolerance in this study (50). Overall, based on the tolerance of gastric and intestinal juices, the LAB strains and *B. subtilis* can act as probiotic strain starters in different fermented food substrates.

**Table 2:** The viable cell counts of the selected LAB strains and *Bacillus subtilis* under simulated gastrointestinal conditions and different heat conditions.

Strain (s)	Simulated gastrointestinal conditions (Log CFU/mL)				Heat tolerance			
	0 h	SGJ (3 h)	SIJ (4 h)	SIJ (8 h)	RT	45°C	85°C	100°C
*L. brevis* DS4-15	7.85 ± 0.01dA	6.25 ± 0.01cA	5.54 ± 0.02bBC	5.44 ± 0.02aA	7.78 ± 0.03bA	7.76 ± 0.01bA	3.25 ± 0.03aC	–
*L. plantarum* AS1-1	8.17 ± 0.01dC	6.65 ± 0.05cA	5.47 ± 0.02bA	5.39 ± 0.04aA	8.80 ± 0.03bB	8.73 ± 0.05bB	2.84 ± 0.03aA	–
*L. plantarum* N2-9	8.18 ± 0.04dC	6.74 ± 0.03cB	5.59 ± 0.04bC	5.50 ± 0.02aA	8.74 ± 0.03cB	8.67 ± 0.03bB	2.94 ± 0.03aAB	–
*L. fermentum* BT2-3	8.10 ± 0.01 dB	6.72 ± 0.01cAB	5.54 ± 0.03bBC	5.43 ± 0.03aA	8.99 ± 0.02cC	8.95 ± 0.05cC	3.76 ± 0.03bD	1.59 ± 0.03aB
*L. fermentum* 8′-2	8.18 ± 0.06dC	6.65 ± 0.04cA	5.49 ± 0.03bAB	5.38 ± 0.03bA	8.80 ± 0.06cB	8.76 ± 0.06cB	3.09 ± 0.07bBC	1.15 ± 0.03aA
*B. subtilis*	8.28 ± 0.04aD	8.26 ± 0.03aC	8.24 ± 0.02aD	8.20 ± 0.02aB	8.57 ± 0.04aB	8.57 ± 0.02aB	8.57 ± 0.03aE	8.56 ± 0.03aC

Mean (*n* = 3) with different lower-case and upper-case letter within each row and column, respectively, for the specific parameter analyzed denotes difference (*p* < 0.05). (−): Not detected. SGJ: Simulated gastric juice. SIJ: Simulated intestinal juice. RT: Room temperature.

### 3.3 Co-culture fermentation of Cili fruit using selected LAB strains and *B. subtilis*

**Figure 1:**
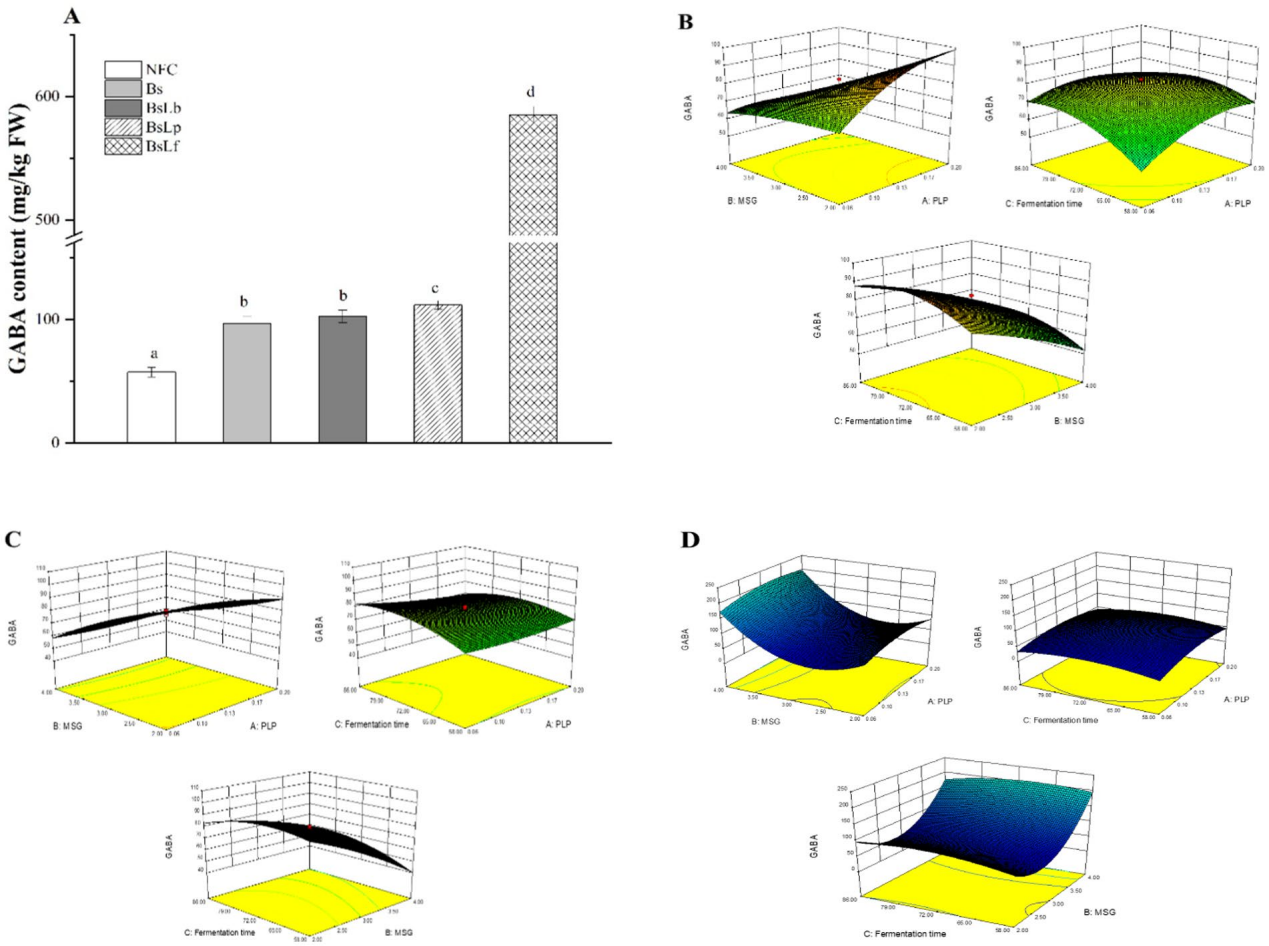
The GABA content in co-cultured fermented Cili fruit substrate at optimized conditions **(A)**. The three-dimensional surface plots showing the influence of different variables on the production of GABA during the co-culture fermentation of Cili fruit substrate using *B. subtilis* and *L. brevis* **(B)**, *B. subtilis* and *L. plantarum* **(C)**, and *B. subtilis* and *L. fermentum* **(D)**.

The preliminary results revealed that upon the co-culture fermentation of Cili fruit using the selected LAB strains and *B. subtilis*, GABA content was only present after the co-culture fermentation of *B. subtilis* + *L. brevis* DS4-15 (BsLb), *B. subtilis* + *L. plantarum* N2-9 (BsLp), and *B. subtilis* + *L. fermentum* BT2-3 (BsLf) of Cili fruit. In fruit substrates, the ability of different microorganisms such as LAB to convert glutamic acid into GABA could be affected by the substrate nutritional composition (e.g., initial glutamate content), fermentation conditions (e.g., pH, temperature and time), and the strain’ ability to adapt to these conditions (26). The independent variables (MSG, PLP and fermentation time) in the fermentation design were selected based on their potential to enhance GABA production, improve the nutritional profile of Cili fruit, and ensure the viability of the probiotic strains used in the fermentation process. To determine feasible ranges, we have consulted published studies on comparable strains or processes (11, 25, 51). In the co-culture fermented Cili fruits, the highest GABA content was observed at 1.32% MSG, 0.13 mM PLP and 72 h fermentation time for BsLb (102.48 mg/kg) and BsLp (111.65 mg/kg), and at 4.68% MSG, 0.13 mM PLP and 72 h fermentation for BsLf (585 mg/kg FW) (Figure 1A). Relative to other studies, the GABA yields in this study (up to 585 mg/kg) was significantly higher than that in other plant-derived substrates, including *B. subtilis* fermented soybeans (11.59–36.21 mg/kg FW) (22), persimmon juice co-fermented with *L. plantarum* C17 and *L. pentosus* Lp-B (216.75 mg/L) (44), and Cili fruit juice fermented by *L. plantarum* NR1-7 (38.5 mg/L) (38.5 mg/L) (17). Moreover, in comparison to unfermented Cili fruit (NFC) and *B. subtilis* (Bs) fermented Cili fruit, co-culture fermentation significantly increased the GABA content in all Cili fruit (Figure 1A). This suggests that the LAB strains could have adapted well to the Cili fruit which resulted in the release of pH lowering metabolites (e.g., organic acids) and modification of the environmental pH suitable for the optimal activities of GABA releasing enzymes such as GAD enzyme during the co-culture fermentation process (52). Also, presence of MSG and PLP in the optimized fermentation conditions acted as co-factors for GAD enzyme activity resulting in the increased synthesis of GABA in the co-culture fermented Cili fruit (BsLf > BsLp > BsLb) (53). These changes were attributed to the effect of strain-specific GAD enzyme activity of the LAB (Table 1) and the synergistic effect of co-culture fermentation with *B. subtilis* of Cili fruit substrate. Similarly, a recent study confirmed an increased GABA content in a culture media enriched with co-factors such as MSG after co-culture fermentation using *Enterococcus faecium* and *Saccharomyces cerevisiae* strains (54). Therefore, at the optimized conditions for BsLf, BsLp, and BsLb, the optimum pH for GAD activity was achieved which enriched the GABA content in the respective co-culture fermented Cili fruits (Figure 1A). This assertion was confirmed by the relationship between the independent and dependent variables on GABA content presented as the 3D response surface plot (Figures 1B–D). In all the plots, the shapes were elliptical, which implied a significant interaction between the variables. Therefore, there existed a synergy during co-culture fermentation of the selected LAB strains and *B. subtilis*, in the presence of MSG and PLP, which enhanced the GABA content of Cili fruit.

### 3.4 Physiochemical properties in co-culture fermentation of Cili fruit using selected LAB strains and *B. subtilis*

#### 3.4.1 Effect of co-culture fermentation on the changes in pH and TTA of Cili fruit

**Figure 2:**
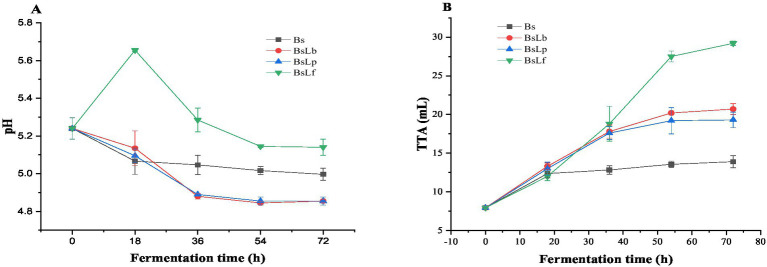
Changes in pH **(A)** and total titratable acidity **(B)** during the co-culture fermentation of Cili fruit. Bs: Fermentation using *B. subtilis*. BsLb: co-culture fermentation using *B. subtilis* and *L. brevis*. BsLp: co-culture fermentation using *B. subtilis* and *L. plantarum*. BsLf: co-culture fermentation using *B. subtilis* and *L. fermentum*.

The changes in pH and TTA values during co-culture fermentation of Cili fruit was shown in Figures 2A,B. Before fermentation, the pH and TTA values of Cili fruit was 5.24 and 7.95 mL, respectively. In all the co-culture fermented Cili fruits, the pH gradually decreased (except for BsLf at 18 h, where it increased, then decreased thereafter), while the TTA values increased with increase in fermentation time. The increase in pH during the initial 18 h of fermentation in BsLF, was likely due to the higher concentration of MSG (4.68%) added to the medium. In published literature, MSG can act as an alkalinizing agent (glutamate ions bind some free H^+^ ions) which may influence pH dynamics in fermentation systems (55). The subsequent decline in pH throughout the extended fermentation period is likely a consequence of the organic acid production by *L. fermentum*. Moreover, compared to Bs sample, significantly lower pH (except BsLf) and higher TTA values were observed during co-culture Cili fruit fermentation. This trend was in agreement with the changes in pH and TTA in several LAB fermented substrate systems (38, 40, 41, 52, 56). In LAB fermented substrates, pH altering metabolites such as organic acids are released during fermentation (57). Therefore, the changes increased acidity due to co-culture fermentation of Cili fruit may positively enhance the release of key enzymes such as esterase, β-glucosidase and GAD, and enhance the bioactive composition, in terms of phytochemicals, soluble fiber and GABA of the end product.

#### 3.4.2 Effect of co-culture fermentation on the changes in key enzymatic activities in Cili fruit

**Figure 3:**
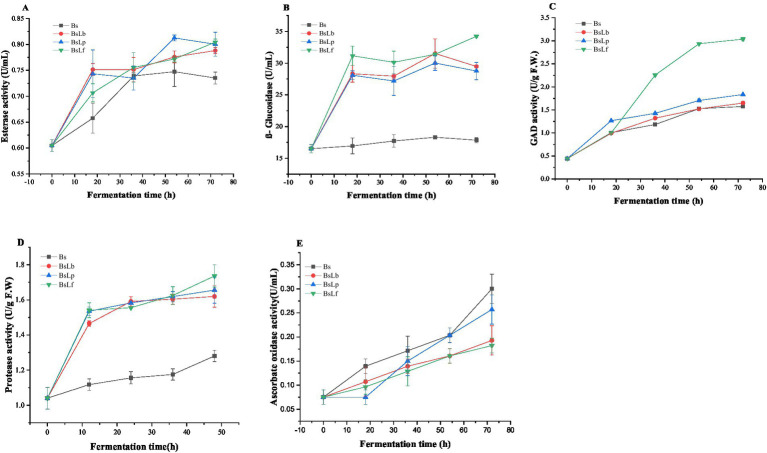
Changes in esterase **(A)**, β-glucosidase **(B)**, glutamate decarboxylase **(C)**, protease **(D)**, and ascorbate oxidase **(E)** enzyme activities during the co-culture fermentation of Cili fruit substrate. Bs: Fermentation using *B. subtilis*. BsLb: co-culture fermentation using *B. subtilis* and *L. brevis*. BsLp: co-culture fermentation using *B. subtilis* and *L. plantarum*. BsLf: co-culture fermentation using *B. subtilis* and *L. fermentum*.

The changes in the activities of esterase, β-glucosidase, GAD, protease and ascorbate oxidase enzymes during the co-culture fermentation of Cili fruit was presented in Figures 3A–E. The results revealed that all the three enzyme activities were detected during the fermentation of Cili fruit: an indication that the microbial starters used adequately adapted and synthesized enzymes as processing aids which can bio-transform substrate components into several bioactive metabolites of functional value after fermentation (58, 59). For esterase enzyme, its activity was 0.60 U/mL for all samples at the start of fermentation, but gradually increased with increased fermentation time to 0.79 U/mL for BsLb, 0.80 U/mL for BsLp and BsLf (Figure 3A). However, compared to Bs, the esterase activity was generally higher in the co-culture fermented Cili fruit. In co-culture fermentations of fruit substrates, the use LAB strain starters was found to enhance the esterase activity during fermentation (60). Due to its mode of action (breakdown of ester bonds, releasing fatty acids and alcohols), increased esterase activity is associated with the release of diverse volatile flavor metabolites (32). For β-glucosidase enzyme, its activity gradually increased with increased fermentation time: the highest increase was observed in BsLf (+107.28%), BsLb (+78.52%), BsLp (+74.16%), and Bs (+8.11%) at the end start of fermentation (Figure 3B). In several studies, enhanced β-glucosidase activities resulted in increased bioactive metabolite contents and functional properties in these substrates (61–64). Similarly, the GAD enzyme activity increased during fermentation of Cili fruit, with the highest observed in BsLf (+583.71%), BsLp (+313.18%), BsLb (+271.57%), and least in Bs (+254.67%) samples (Figure 3C). The changes in GAD activity during fermentation could potentially increase the GABA production in the Cili fruit (65). The protease activity increased with the increasing fermentation time (Figure 3D). However, compared with *B. subtilis* only, *B. subtilis* co- cultured with LAB exhibited the significantly higher protease activities. Specifically, protease activity increased from 1.04 U/mL to 1.28, 1.61, 1.65, and 1.73 U/mL, respectively, for Bs, BsLb, BsLp, and BsLf (Figure 3D). Our findings, along with a previous study by Ren et al. (66) underscore the potential of co-fermenting *B. subtilis* and LAB to enhance extracellular protease activity. Moreover, co-culture fermented Cili fruit have reduced the ascorbate oxidase enzyme activity (Figure 3E) and had a stabilizing effect on the vitamin C content of these samples. Furthermore, the ascorbate oxidase activity also showed a continuous increase throughout the fermentation process. However, in the co-cultured Cili fruit, the AAO activity was relatively lower (0.19, 0.25, and 0.18 U/mL for the co-cultures with BsLb, BsLp, and BsLf, respectively) compared to that observed in monoculture fermentation (0.3 U/mL) (Figure 3E). This was due to the production of organic acid by LAB, which leads to a reduction in pH and hence downregulates the activity of ascorbate oxidase in the fermentates (67, 68).

### 3.5 Effect of co-culture fermentation on the bioactive composition of Cili fruit

#### 3.5.1 Vitamin C content

The results of the vitamin C content after co-culture fermentation were presented in Table 3. Before fermentation (NFC), the Vitamin C content was 2,081.74 mg/kg FW, which decreased in Bs (−67.64%), BsLb (−41.50%), BsLp (−64.43%), and BsLf (−53.86%) at the end of fermentation. These findings were in agreement with a recent study where the vitamin C and L-ascorbic acid contents in leaves significantly decreased after aerobic and anaerobic solid-state fermentation (69). The vitamin C decrease may be attributed to the increased activity of ascorbate oxidase enzyme produced by the microbial starters used during fermentation (70). On the other hand, relative to Bs (673.61 mg/kg FW), the vitamin C content was higher in co-culture fermented Cili fruit, especially in BsLb (+80.94%), BsLf (42.60%), and BsLp (+9.93%) (Table 3). This may be attributed to its sensitivity to oxidation (71), as the monoculture fermentation was carried out under aerobic conditions. Conversely, in the co-culture setup, lactic acid bacteria could inhibit the oxygen utilization of *B. subtilis*, creating an anaerobic environment that helps reduce the oxidation of vitamin C. In an earlier study, organic acids produced during LAB fermentation were found to stabilize the ascorbic acid content in Amaranthus paste after LAB fermentation (68). Hence, the higher acidification rate (Figures 2A,B) in co-culture fermented Cili fruit could have reduced the ascorbate oxidase enzyme activity and had a stabilizing effect on vitamin C content of these samples. The improved vitamin C content of the co-culture fermented Cili fruit may significantly enhance its functional properties (2).

**Table 3:** The bioactive composition and functional properties of co-culture fermented Cili fruit.

Sample (s)	Bioactive component			Functional property				
				Antioxidant activity			Anti-hangover activity	
	Vitamin C (mg/kg FW)	TPC (mg GAE/g)	TFC (mg QE/g)	DPPH (%)	ABTS (%)	FRAP (Fe^2+^ mM)	ADH activity (%)	Hydroxyl inhibition ability (%)
NFC	2,081.74 ± 30.57e	153.28 ± 10.58a	125.51 ± 14.05a	90.18 ± 1.38^a^	82.71 ± 1.10^a^	2.51 ± 0.15^a^	35.22 ± 7.47^a^	9.19 ± 1.67^a^
Bs	673.61 ± 7.53a	251.16 ± 9.96b	132.96 ± 12.11ab	95.49 ± 0.21^b^	85.43 ± 0.25^b^	2.86 ± 0.01^b^	47.85 ± 5.90^ab^	12.57 ± 0.90^b^
BsLb	1,217.84 ± 24.64d	259.85 ± 1.62b	172.33 ± 13.74d	95.89 ± 0.10^b^	87.38 ± 0.22^bc^	3.04 ± 0.02^b^	55.98 ± 3.15^bc^	12.21 ± 0.16^b^
BsLp	740.48 ± 9.36b	256.00 ± 8.60b	155.24 ± 6.57 cd	95.71 ± 0.05^b^	87.38 ± 0.22^bc^	3.04 ± 0.01^b^	56.05 ± 8.64^bc^	15.85 ± 1.02^c^
BsLf	960.59 ± 24.64c	275.17 ± 1.70c	140.54 ± 4.94bc	96.18 ± 0.03^b^	88 ± 0.22^c^	3.04 ± 0.03^b^	64.04 ± 3.87^c^	14.90 ± 0.92^bc^

Mean (*n* = 3) with different lower-case letter within each column denotes difference (*p* < 0.05). NFC: Non fermented Cili fruit. Bs: Fermentation using *B. subtilis*. BsLb: co-culture fermentation using *B. subtilis* and *L. brevis*. BsLp: co-culture fermentation using *B. subtilis* and *L. plantarum*. BsLf: co-culture fermentation using *B. subtilis* and *L. fermentum*.

#### 3.5.2 Total phenolic (TPC) and flavonoid (TFC) content

The TPC and TFC content in the different samples was were presented in Table 3. The results showed that compared to NFC (TPC: 153.28 mg GAE/g DW, TFC: 125.51 mg QE/g DW), the TPC and TFC values significantly increased after fermentation. For TPC, the highest increase was found in BsLf (+79.52%), then BsLb (+69.53%), BsLp (+67.02%) and Bs (+63.86%), while the TFC was highest in BsLb (+37.30%), then BsLp (+23.68%), BsLf (+11.97%), and Bs (+5.93%) (Table 3). The increase in TPC and TFC observed in this study was in agreement with several studies where LAB fermentation consistently increased the phytochemical content (TPC, TFC) of the respective substrates (56, 72, 73). Moreover, the TPC and TFC content of lentils significantly increased after co-fermentation *Lactobacillus* spp. and *Bacillus subtilis* (74). As shown in Figure 3, the increased activity of esterase (Figure 3A) and β-glucosidase (Figure 3B) enzymes during Cili fruit fermentation could have facilitated the hydrolysis and release of bound phytochemicals, thereby, increasing the TPC and TFC content after fermentation (75). Increased content of phytochemicals in substrates are positively associated with improved antioxidant properties (2).

#### 3.5.3 Free amino acid (FAA) composition

The composition of 17 FAA in the co-culture fermented Cili fruit was presented in Table 4. The results showed that with the exception of Arg, Try, and Cyst, the content of the remaining 14 FAA generally increased in fermented Cili fruit samples. Subsequently, the total FAA content was highest in BsLf (6,191.39 mg/kg FW), Bs (2,423.87 mg/kg FW), BsLb (2,278.53 mg/Kg FW) and BsLp (2,278.37 mg/kg FW) relative to NFC (1,124.59 mg/kg FW). In many published studies, FAA content has been reported in single and/or mixed LAB strain fermented substrates (76). Similar to the increased GABA content (Figure 1A) due to optimal pH for GAD activity (Figure 3C), the pH during and after fermentation could have favored synthesis of other strain-specific proteolytic enzymes, which degraded proteins and peptides in the Cili fruit into free amino acids during and/or after substrate fermentation (77).

**Table 4:** The free amino acid composition of co-culture fermented Cili fruit and its control samples.

FAA (mg/kg FW)	NFC	Bs	BsLb	BsLp	BsLf
Asp	48.97 ± 22.45^b^	11.1 ± 0.64^a^	69.33 ± 5.80^b^	72.30 ± 1.12^b^	102.07 ± 4.81^c^
Glu	14.49 ± 0.00^a^	2,259.71 ± 21.72^b^	2,098.27 ± 50.96^b^	2,084.65 ± 215.96^b^	6,013.35 ± 105.51^c^
Ser	2.69 ± 0.87^a^	2.48 ± 0.55^a^	4.43 ± 0.34^b^	4.47 ± 0.38^b^	2.12 ± 0.43^a^
His	1.44 ± 0.31^a^	4.47 ± 0.54^c^	3.13 ± 0.80^bc^	3.20 ± 0.64^c^	1.71 ± 0.84^ab^
Gly	1.35 ± 0.83^a^	7.07 ± 0.98^b^	4.54 ± 1.75^ab^	5.13 ± 2.95^ab^	3.64 ± 1.32^ab^
Thr	1.24 ± 1.18^a^	5.61 ± 0.14^b^	3.57 ± 1.22^ab^	4.15 ± 1.31^ab^	5.28 ± 1.77^b^
Arg	9.70 ± 4.33^b^	2.53 ± 0.67^a^	3.09 ± 1.38^a^	3.70 ± 1.65^a^	2.97 ± 1.07^a^
Ala	10.99 ± 4.81^a^	59.27 ± 3.92^c^	38.19 ± 10.00^bc^	41.28 ± 14.28^bc^	30.89 ± 9.89^ab^
Tyr	1.32 ± 0.13^b^	0.66 ± 0.09^ab^	0.76 ± 0.28^ab^	0.58 ± 0.51^a^	0.11 ± 0.07^a^
Cyst	0.01 ± 0.01^a^	0.32 ± 0.29^a^	0.01 ± 0.01^a^	0.01 ± 0.01^a^	0.02 ± 0.01^a^
Val	0.27 ± 0.14^a^	1.56 ± 0.15^c^	0.97 ± 0.02^b^	1.08 ± 0.04^b^	0.26 ± 0.09^a^
Met	2.15 ± 0.05^a^	3.56 ± 2.07^b^	0.52 ± 0.07^a^	0.50 ± 0.09^a^	1.05 ± 0.15^a^
Phe	2.18 ± 0.74^a^	8.05 ± 1.25^b^	6.63 ± 0.30^b^	6.85 ± 0.24^b^	2.23 ± 1.21^a^
Ile	1.81 ± 0.80^a^	6.44 ± 0.01^c^	4.80 ± 0.34^b^	5.37 ± 0.67^bc^	1.15 ± 0.64^a^
Leu	0.04 ± 0.00^a^	13.07 ± 1.08^c^	7.69 ± 1.05^b^	9.18 ± 2.57^b^	2.72 ± 0.30^a^
Lys	1.04 ± 0.54^a^	5.17 ± 0.09^a^	2.95 ± 2.29^a^	3.7 ± 2.58^a^	2.28 ± 2.11^a^
Pro	12.29 ± 5.65^a^	9.84 ± 2.53^a^	13.79 ± 0.42^a^	17.02 ± 3.07^a^	15.73 ± 1.03^a^
Total	124.59 ± 40.11^a^	2,423.87 ± 20.70^b^	2,278.53 ± 60.57^b^	2,278.37 ± 242.97^b^	6,191.39 ± 126.87^c^

Mean (*n* = 3) with different lower-case letter within each row denotes difference (*p* < 0.05). NFC: Non fermented Cili fruit. Bs: Fermentation using *B. subtilis*. BsLb: co-culture fermentation using *B. subtilis* and *L. brevis*. BsLp: co-culture fermentation using *B. subtilis* and *L. plantarum*. BsLf: co-culture fermentation using *B. subtilis* and *L. fermentum*.

### 3.6 Effect of co-culture fermentation on the antioxidant activity and anti-hangover activity of Cili fruit

To determine the effect of co-culture fermentation on the functional properties of Cili fruit, the antioxidant activity based on the DPPH, ABTS, and FRAP capacity, and the anti-hangover activity based on alcohol dehydrogenase (ADH) and hydroxyl inhibition ability were reported on (Table 3).

For the antioxidant activity, the results showed that compared to NFC, co-culture fermentation significantly increased all the 3 measured properties, including DPPH (from 90.18% to 95.49–96.18%), ABTS (from 82.71% to 85.43–88.00%), and FRAP (from 2.51 to 3.04%) activity in the Cili fruit. Also, higher values of the 3 antioxidant parameters were observed in co-culture fermented Cili fruit substrate than in Bs (Table 3). The increase in bioactive metabolites such as GABA and phenolics has been associated with improved antioxidant activity (78, 79). As shown in Figure 1A and Table 3, the increased content of GABA and phenolic compounds, especially in co-culture followed by Bs and least in NFC could have had positively influenced the antioxidant activity of the samples.

Like the antioxidant activity, a similar trend was observed in the results of the anti-hangover activity of the Cili fruit substrate after co-culture fermentation when compared to NFC and Bs samples (Table 3). For instance, the ADH, which plays a crucial role in ethanol metabolism and its increased activity associated with alleviating hangover symptoms, increased from 35.22% in NFC to 47.85, 55.98, 56.05, and 64.04%, in Bs, BsLb, BsLp, and BsLf, respectively. Previous studies indicate a significant relationship between GABA (80–82) and phenolic compounds (83) in food and ADH enzyme activity. However, further comprehensive research is essential to investigate other anti-hangover bioactive constituents in fermented foods, beyond GABA and phenolic compounds. On the other hand, the hydroxyl inhibition ability, an indicator of the ability to reduce the increased of acetaldehyde levels and oxidative damage due to reactive oxygen species (e.g., hydroxyl radicals) due to excessive alcohol consumption (84), significantly increased from 9.19% in NFC to 12.57, 12.21, 15.85, and 14.90%, in Bs, BsLb, BsLp, and BsLf, respectively (Table 3). These results suggests that co-culture fermentation enhanced the ADH and hydroxyl inhibition activity of Cili fruit. The synergistic effect of co-culture fermentation evidently enhanced the content of bioactive metabolites such as GABA and phytochemicals (TPC, TFC), which are reported to facilitate ethanol metabolism and potentially mitigating the adverse effects of alcohol consumption (85, 86).

## 4 Conclusion

In this study, 3 (*L. brevis* DS4-15*, L. plantarum* N2-9, and *L. fermentum* BT2-3) of the 20 LAB strains successfully enriched the GABA content in Cili fruit after co-culture fermentation at the optimized conditions. At the optimized fermentation conditions, higher GABA contents were found in co-culture fermented Cili fruit (highest in *B. subtilis* and *L. fermentum*: 585 mg/kg) than in *B. subtilis* fermented or unfermented Cili fruit. Furthermore, the bioactive compounds such as total phenols, total flavonoids and free amino acids were generally higher after co-culture fermentation of Cili fruit: this could have led to the observed improvement of the antioxidant activity and anti-hangover properties in these samples. The changes observed were attributed to the ability of the LAB strain to alter the pH during co-culture fermentation with *B. subtilis* resulting in a synergistic effect which promoted and increased enzyme activities of esterase, β-glucosidase, and GAD during co-culture fermentation. This subsequently increased the release of bioactive metabolites in Cili fruit and enhanced its nutritional and functional properties. These findings revealed that there was a synergy in co-culture fermentation which improved the bioactive and functional properties of Cili fruit as a novel GABA enriched ingredient for potential use in the food industry.

## Data Availability

The raw data supporting the conclusions of this article will be made available by the authors without undue reservation.
