# Supplementary material for: Screening of LAB strains and their co-culture fermentation with Bacillus subtilis of Cili fruit substrate: impact on γ-aminobutyric acid enrichment, key enzyme activities, bioactive and functional properties
Source: Front Nutr. 2025 Jul 4;12:1622745. doi: 10.3389/fnut.2025.1622745 (PMC12271109; doi:10.3389/fnut.2025.1622745)
Supplement: Supplementary file 1 [file Table_1.docx]

SUPPLEMENTARY TABLE S1 Design and results of the Central Composite Design experiment on GABA content in co-culture fermented Cili fruit substrate

|  |  |  |  | **GABA yield (mg/mL)** |  |  |
| --- | --- | --- | --- | --- | --- | --- |
| **Run** | **A: PLP (mM)** | **B: MSG (%)** | **C: Fermentation time (h)** | **BsLb** | **BsLp** | **BsLf** |
| 1 | 0.01 | 3 | 72 | 60.61 | 60.61 | 65.11 |
| 2 | 0.06 | 4 | 58 | 42.76 | 42.76 | 55.78 |
| 3 | 0.13 | 3 | 72 | 78.18 | 78.18 | 73.35 |
| 4 | 0.13 | 1.32 | 72 | 98.89 | 98.89 | 123.62 |
| 5 | 0.2 | 4 | 58 | 40.58 | 40.58 | 94.62 |
| 6 | 0.06 | 2 | 58 | 59.12 | 59.12 | 75.55 |
| 7 | 0.2 | 4 | 86 | 27.84 | 27.84 | 75.87 |
| 8 | 0.06 | 2 | 86 | 63.21 | 63.21 | 105.39 |
| 9 | 0.13 | 3 | 48.45 | 47.08 | 47.08 | 83.35 |
| 10 | 0.06 | 4 | 86 | 52.93 | 52.93 | 56.46 |
| 11 | 0.13 | 3 | 72 | 77.98 | 77.98 | 70.05 |
| 12 | 0.2 | 2 | 86 | 82.77 | 82.77 | 114.39 |
| 13 | 0.13 | 3 | 72 | 79.06 | 79.06 | 88.88 |
| 14 | 0.25 | 3 | 72 | 76.11 | 76.11 | 78.97 |
| 15 | 0.13 | 3 | 95.55 | 84.91 | 84.91 | 63.21 |
| 16 | 0.13 | 3 | 72 | 82.59 | 82.59 | 71.79 |
| 17 | 0.13 | 3 | 72 | 79.08 | 79.08 | 69.54 |
| 18 | 0.2 | 2 | 58 | 93.14 | 93.14 | 83.01 |
| 19 | 0.13 | 4.68 | 72 | 66.691 | 66.69 | 580.11 |
| 20 | 0.13 | 3 | 72 | 78.82 | 78.82 | 81.32 |

BsLb: co-culture fermentation using *B. subtilis* and *L. brevis*. BsLp: co-culture fermentation using *B. subtilis* and *L. plantarum*. BsLf: co-culture fermentation using *B. subtilis* and *L. fermentum*.
