# Supplementary material for: Screening of LAB strains and their co-culture fermentation with Bacillus subtilis of Cili fruit substrate: impact on γ-aminobutyric acid enrichment, key enzyme activities, bioactive and functional properties
Source: Front Nutr. 2025 Jul 4;12:1622745. doi: 10.3389/fnut.2025.1622745 (PMC12271109; doi:10.3389/fnut.2025.1622745)
Supplement: Supplementary file 2 [file Image_1.pdf]

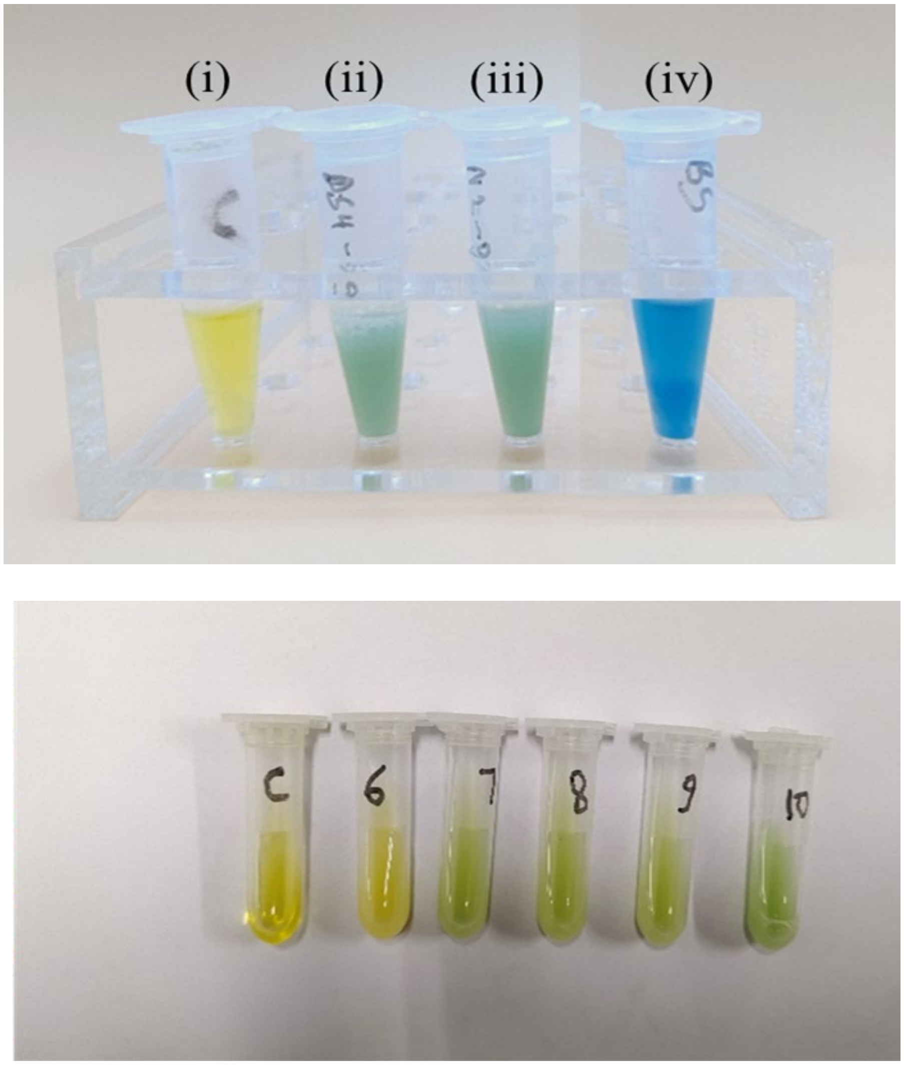

Figure S1. The representative image displays selected strains exhibiting low (ii), medium (iii), and high (iv) levels of GAD activity during a rapid colorimetric assay utilizing bromocresol green as a pH indicator for GAD activity. Negative results and the control stayed yellow(i), exhibiting no color change.
